# 2-[3-Cyano-4-(2-methyl­prop­oxy)phen­yl]-4-methyl­thia­zole-5-carboxylic acid pyridine solvate

**DOI:** 10.1107/S1600536809039002

**Published:** 2009-10-03

**Authors:** Xiong Zhu, Yue Wang, Tao Lu

**Affiliations:** a Institute of Medicine, China Pharmaceutical University, Nanjing 210009, People’s Republic of China; b College of Basic Sciences, China Pharmaceutical University, Nanjing 210009, People’s Republic of China

## Abstract

In the title compound, C_16_H_16_N_2_O_3_S·C_5_H_5_N, the benzene and thia­zole rings of the Febuxostat [2-(3-cyano-4-isobut­yloxy)phenyl-4-methyl-5-thia­zolecarboxylic acid] mol­ecule are almost coplanar [dihedral angle = 2.4 (1)°]. The carboxyl group is coplanar with the thia­zole ring [O—C—C—C and O—C—C—S torsion angles of −0.7 (4) and 0.6 (3)°, respectively]. The pyridine mol­ecule of crystallization is linked to the Febuxostat mol­ecule through an O—H⋯N hydrogen bond. A weak π–π stacking inter­action is observed between the benzene ring of the Febuxostat mol­ecule and pyridine mol­ecule, with a centroid–centroid distance of 3.7530 (18) Å.

## Related literature

For general background to gout, see: Alexander (2008 ▶). For the synthesis, polymorphism, stability and biological activity of Febuxostat, see: Edwards (2009 ▶); Hiramatsu *et al.* (2000 ▶); Perez-Ruiz *et al.* (2008 ▶); Sorbera *et al.* (2001 ▶); Zhou *et al.* (2007 ▶). For a related structure, see: Fontrodona *et al.* (2001 ▶). 
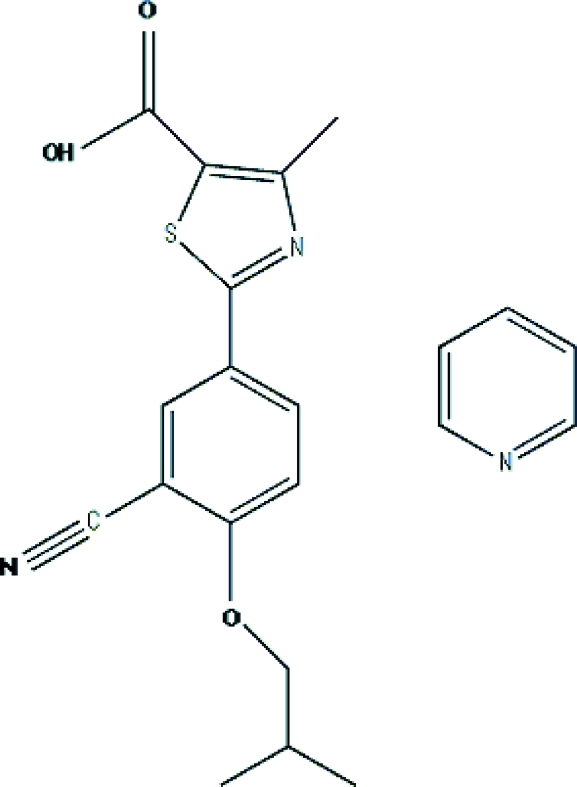


## Experimental

### 

#### Crystal data

C_16_H_16_N_2_O_3_S·C_5_H_5_N
*M* *_r_* = 395.47
Triclinic, 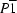

*a* = 8.6040 (17) Å
*b* = 10.339 (2) Å
*c* = 12.611 (3) Å
α = 82.51 (3)°
β = 80.69 (3)°
γ = 69.61 (3)°
*V* = 1034.4 (4) Å^3^
*Z* = 2
Mo *K*α radiation
μ = 0.18 mm^−1^
*T* = 296 K
0.30 × 0.20 × 0.20 mm

#### Data collection

Enraf–Nonius CAD-4 diffractometer
Absorption correction: ψ scan (North *et al.*, 1968 ▶) *T*_min_ = 0.947, *T*_max_ = 0.964
4017 measured reflections
3747 independent reflections
2815 reflections with *I* > 2σ(*I*)
*R*_int_ = 0.032
3 standard reflections every 200 reflections intensity decay: 1%

#### Refinement

*R*[*F* ^2^ > 2σ(*F* ^2^)] = 0.049
*wR*(*F* ^2^) = 0.156
*S* = 0.99
3747 reflections
255 parameters
H-atom parameters constrained
Δρ_max_ = 0.33 e Å^−3^
Δρ_min_ = −0.19 e Å^−3^

### 

Data collection: *CAD-4 EXPRESS* (Enraf–Nonius, 1994 ▶); cell refinement: *CAD-4 EXPRESS*; data reduction: *XCAD4* (Harms & Wocadlo,1995 ▶); program(s) used to solve structure: *SHELXS97* (Sheldrick, 2008 ▶); program(s) used to refine structure: *SHELXL97* (Sheldrick, 2008 ▶); molecular graphics: *SHELXTL* (Sheldrick, 2008 ▶); software used to prepare material for publication: *SHELXL97*.

## Supplementary Material

Crystal structure: contains datablocks I, global. DOI: 10.1107/S1600536809039002/ci2885sup1.cif

Structure factors: contains datablocks I. DOI: 10.1107/S1600536809039002/ci2885Isup2.hkl

Additional supplementary materials:  crystallographic information; 3D view; checkCIF report

## Figures and Tables

**Table 1:** Hydrogen-bond geometry (Å, °)

*D*—H⋯*A*	*D*—H	H⋯*A*	*D*⋯*A*	*D*—H⋯*A*
O2—H2*A*⋯N3^i^	0.82	1.79	2.611 (3)	174

Symmetry code: (i) 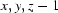.

**Fig. 1.**
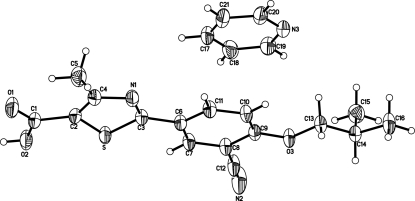
The molecular structure of the title compound, showing 50% probability displacement ellipsoids.

## supplementary crystallographic information

### Comment

The oxidation of xanthine results in the formation of uric acid. Disorders of uric acid metabolism include gout which is the most common inflammatory arthritis initiated by tissue deposition of monosodium urate (MSU) crystals (Alexander, 2008). Some inventions are related to methods of preserving or increasing renal function in a subject by administering a therapeutically effective amount of at least one xanthine oxidoreductase inhibiting compound. 2-(3-Cyano-4-isobutyloxy)phenyl-4-methyl-5-thiazolecarboxylic acid (Febuxostat) is one of the novel drug that have been evaluated and shown to be highly effective in the management of hyperuricemia (Perez-Ruiz *et al.*, 2008; Edwards, 2009), thus enlarging the therapeutic options available to lower uric acid levels. Many patents or papers have been reported on the synthesis, polymorphism and their effect on the stability and bioavailability of this drug (Hiramatsu *et al.*, 2000; Sorbera *et al.*, 2001; Zhou *et al.*, 2007). However, there are few reports on its single-crystal structure. In the present study, we report the crystal structure of the title compound.

The asymmetric unit of the title compound contains one febuxostat molecule and one pyridine molecule. The phenyl ring and thiazole rings of the febuxostat molecule are almost coplanar (Fig. 1), with the dihedral angle between them being 2.4 (1)°. The carboxyl group is coplanar with the thiazole ring as indicated by torsion angles O1—C1—C2—C4 and O2—C1—C2—S of -0.7 (4)° and 0.6 (3)°, respectively. Bond lengths and angles are comparable to those observed in a related structure (Fontrodona *et al.*, 2001).

In the crystal, pyridine moleclue is linked to the febuxostat molecule through a O2—H2A···N3(*x*, *y*, 1 + *z*) hydrogen bond (Table 1). A weak π-π stacking interaction is observed between the benzene ring and pyridine molecule, with a centroid-to-centroid distance of 3.7530 (18) Å.

### Experimental

2-(3-Formyl-4-isobutoxyphenyl)-4-methylthiazole-5-carboxylic acid ethyl ester (42.6 g, 0.123 mol) was treated with formic acid (384 ml), sodium formate (15.3 g, 0.147 mol) and hydroxylamine hydrochloride (10.2 g, 0.147 mol) to give 21.4 g of 2-(3-cyano-4-isobutoxyphenyl)-4-methylthiazole-5-carboxylic acid ethyl ester (yield 50.5%). Then it was hydrolyzed with NaOH in tetrahydrofuran and ethanol. Finally, brown block crystals of the title compound appropriate for X-ray data collection were obtained by slow evaporation of a pyridine solution at room temperature (yield 70%).

### Refinement

All H atoms were initially located from a difference Fourier map and then were regenerated at ideal positions and treated as riding, with O-H = 0.82 Å, C-H = 0.93-0.98 Å and *U*_iso_(H) = 1.2-1.5*U*_eq_(C,O).

### Crystal data

**Table:**

C_16_H_16_N_2_O_3_S·C_5_H_5_N	*Z* = 2
*M**_r_* = 395.47	*F*(000) = 416
Triclinic, *P*1	*D*_x_ = 1.270 Mg m^−^^3^
Hall symbol: -P 1	Mo *K*α radiation, λ = 0.71073 Å
*a* = 8.6040 (17) Å	Cell parameters from 25 reflections
*b* = 10.339 (2) Å	θ = 10–13°
*c* = 12.611 (3) Å	µ = 0.18 mm^−^^1^
α = 82.51 (3)°	*T* = 296 K
β = 80.69 (3)°	Block, brown
γ = 69.61 (3)°	0.30 × 0.20 × 0.20 mm
*V* = 1034.4 (4) Å^3^	

### Data collection

**Table:**

Enraf–Nonius CAD-4 diffractometer	2815 reflections with *I* > 2σ(*I*)
Radiation source: fine-focus sealed tube	*R*_int_ = 0.032
graphite	θ_max_ = 25.3°, θ_min_ = 1.6°
ω/2θ scans	*h* = 0→10
Absorption correction: ψ scan (North *et al.*, 1968)	*k* = −11→12
*T*_min_ = 0.947, *T*_max_ = 0.964	*l* = −14→15
4017 measured reflections	3 standard reflections every 200 reflections
3747 independent reflections	intensity decay: 1%

### Refinement

**Table:**

Refinement on *F*^2^	Secondary atom site location: difference Fourier map
Least-squares matrix: full	Hydrogen site location: inferred from neighbouring sites
*R*[*F*^2^ > 2σ(*F*^2^)] = 0.049	H-atom parameters constrained
*wR*(*F*^2^) = 0.156	*w* = 1/[σ^2^(*F*_o_^2^) + (0.1*P*)^2^ + 0.12*P*] where *P* = (*F*_o_^2^ + 2*F*_c_^2^)/3
*S* = 0.99	(Δ/σ)_max_ = 0.001
3747 reflections	Δρ_max_ = 0.33 e Å^−^^3^
255 parameters	Δρ_min_ = −0.19 e Å^−^^3^
0 restraints	Extinction correction: *SHELXL97* (Sheldrick, 2008), Fc^*^=kFc[1+0.001xFc^2^λ^3^/sin(2θ)]^-1/4^
Primary atom site location: structure-invariant direct methods	Extinction coefficient: 0.030 (5)

### Special details

**Table:**

Geometry. All e.s.d.'s (except the e.s.d. in the dihedral angle between two l.s. planes) are estimated using the full covariance matrix. The cell e.s.d.'s are taken into account individually in the estimation of e.s.d.'s in distances, angles and torsion angles; correlations between e.s.d.'s in cell parameters are only used when they are defined by crystal symmetry. An approximate (isotropic) treatment of cell e.s.d.'s is used for estimating e.s.d.'s involving l.s. planes.
Refinement. Refinement of *F*^2^ against ALL reflections. The weighted *R*-factor *wR* and goodness of fit *S* are based on *F*^2^, conventional *R*-factors *R* are based on *F*, with *F* set to zero for negative *F*^2^. The threshold expression of *F*^2^ > σ(*F*^2^) is used only for calculating *R*-factors(gt) *etc*. and is not relevant to the choice of reflections for refinement. *R*-factors based on *F*^2^ are statistically about twice as large as those based on *F*, and *R*- factors based on ALL data will be even larger.

### Fractional atomic coordinates and isotropic or equivalent isotropic displacement parameters (Å^2^)

**Table:**

	*x*	*y*	*z*	*U*_iso_*/*U*_eq_	
S	0.83182 (8)	0.26459 (7)	0.30078 (4)	0.0564 (2)	
O1	0.5471 (2)	0.2112 (2)	0.10983 (14)	0.0754 (6)	
O2	0.7344 (2)	0.3198 (2)	0.08925 (14)	0.0754 (6)	
H2A	0.7091	0.3381	0.0277	0.113*	
O3	1.1361 (2)	0.20514 (18)	0.76698 (12)	0.0586 (5)	
N1	0.6870 (3)	0.1219 (2)	0.43509 (15)	0.0586 (5)	
N2	1.2786 (4)	0.4042 (4)	0.5613 (2)	0.1041 (10)	
C1	0.6483 (3)	0.2464 (3)	0.14487 (19)	0.0570 (6)	
C2	0.6882 (3)	0.2095 (3)	0.25744 (18)	0.0523 (6)	
C3	0.7976 (3)	0.1847 (2)	0.42631 (17)	0.0510 (6)	
C4	0.6240 (3)	0.1354 (3)	0.33987 (18)	0.0562 (6)	
C5	0.4943 (4)	0.0713 (3)	0.3353 (2)	0.0790 (9)	
H5A	0.4167	0.1282	0.2874	0.118*	
H5B	0.4357	0.0636	0.4062	0.118*	
H5C	0.5472	−0.0191	0.3096	0.118*	
C6	0.8868 (3)	0.1864 (2)	0.51606 (17)	0.0509 (6)	
C7	0.9984 (3)	0.2585 (3)	0.50346 (18)	0.0554 (6)	
H7A	1.0183	0.3054	0.4374	0.067*	
C8	1.0807 (3)	0.2614 (2)	0.58894 (17)	0.0529 (6)	
C9	1.0521 (3)	0.1928 (2)	0.68921 (17)	0.0494 (5)	
C10	0.9425 (3)	0.1192 (2)	0.70122 (17)	0.0539 (6)	
H10A	0.9230	0.0714	0.7669	0.065*	
C11	0.8622 (3)	0.1166 (3)	0.61544 (18)	0.0555 (6)	
H11A	0.7892	0.0664	0.6247	0.067*	
C12	1.1917 (4)	0.3398 (3)	0.57499 (19)	0.0705 (8)	
C13	1.1139 (3)	0.1363 (3)	0.87244 (17)	0.0532 (6)	
H13A	1.1489	0.0371	0.8677	0.064*	
H13B	0.9971	0.1684	0.9025	0.064*	
C14	1.2185 (3)	0.1690 (3)	0.94322 (18)	0.0555 (6)	
H14A	1.3341	0.1432	0.9081	0.067*	
C15	1.1568 (4)	0.3222 (3)	0.9600 (2)	0.0716 (7)	
H15A	1.1603	0.3744	0.8914	0.107*	
H15B	1.2270	0.3404	1.0039	0.107*	
H15C	1.0440	0.3487	0.9952	0.107*	
C16	1.2148 (4)	0.0832 (4)	1.0511 (2)	0.0829 (9)	
H16A	1.2555	−0.0134	1.0389	0.124*	
H16B	1.1021	0.1077	1.0865	0.124*	
H16C	1.2844	0.1012	1.0956	0.124*	
N3	0.6706 (3)	0.3879 (3)	0.89010 (17)	0.0662 (6)	
C17	0.5872 (4)	0.4524 (3)	0.6832 (2)	0.0784 (9)	
H17A	0.5589	0.4742	0.6134	0.094*	
C18	0.6965 (4)	0.5024 (3)	0.7160 (2)	0.0755 (8)	
H18A	0.7444	0.5589	0.6687	0.091*	
C19	0.7359 (4)	0.4686 (3)	0.8200 (2)	0.0702 (7)	
H19A	0.8106	0.5035	0.8418	0.084*	
C20	0.5646 (3)	0.3398 (3)	0.8567 (2)	0.0743 (8)	
H20A	0.5182	0.2830	0.9048	0.089*	
C21	0.5200 (4)	0.3700 (4)	0.7544 (2)	0.0816 (9)	
H21A	0.4447	0.3345	0.7341	0.098*	

### Atomic displacement parameters (Å^2^)

**Table:**

	*U*^11^	*U*^22^	*U*^33^	*U*^12^	*U*^13^	*U*^23^
S	0.0649 (4)	0.0694 (4)	0.0399 (3)	−0.0261 (3)	−0.0190 (3)	0.0038 (3)
O1	0.0859 (13)	0.1005 (15)	0.0542 (11)	−0.0423 (11)	−0.0318 (9)	0.0028 (10)
O2	0.0786 (12)	0.1131 (16)	0.0442 (9)	−0.0428 (12)	−0.0256 (9)	0.0128 (10)
O3	0.0751 (11)	0.0709 (11)	0.0383 (8)	−0.0323 (9)	−0.0229 (7)	0.0072 (7)
N1	0.0607 (12)	0.0784 (14)	0.0422 (10)	−0.0284 (11)	−0.0138 (9)	−0.0006 (9)
N2	0.148 (3)	0.140 (3)	0.0683 (16)	−0.102 (2)	−0.0507 (17)	0.0359 (16)
C1	0.0562 (14)	0.0680 (16)	0.0436 (12)	−0.0115 (12)	−0.0179 (11)	−0.0042 (11)
C2	0.0498 (13)	0.0645 (15)	0.0428 (12)	−0.0140 (11)	−0.0157 (10)	−0.0078 (10)
C3	0.0547 (13)	0.0588 (14)	0.0387 (12)	−0.0158 (11)	−0.0124 (10)	−0.0011 (10)
C4	0.0553 (14)	0.0725 (16)	0.0445 (12)	−0.0224 (12)	−0.0131 (10)	−0.0058 (11)
C5	0.0804 (19)	0.113 (2)	0.0605 (16)	−0.0508 (18)	−0.0194 (14)	−0.0009 (16)
C6	0.0562 (13)	0.0583 (14)	0.0383 (11)	−0.0164 (11)	−0.0130 (10)	−0.0027 (10)
C7	0.0665 (15)	0.0631 (15)	0.0382 (11)	−0.0226 (12)	−0.0155 (10)	0.0049 (10)
C8	0.0653 (15)	0.0591 (14)	0.0391 (12)	−0.0249 (12)	−0.0163 (10)	0.0034 (10)
C9	0.0599 (13)	0.0503 (13)	0.0378 (11)	−0.0152 (11)	−0.0148 (10)	−0.0015 (9)
C10	0.0697 (15)	0.0583 (14)	0.0362 (11)	−0.0240 (12)	−0.0147 (10)	0.0053 (10)
C11	0.0658 (15)	0.0625 (15)	0.0448 (12)	−0.0273 (12)	−0.0150 (10)	−0.0011 (10)
C12	0.095 (2)	0.089 (2)	0.0439 (13)	−0.0501 (17)	−0.0309 (13)	0.0172 (13)
C13	0.0673 (15)	0.0578 (14)	0.0367 (11)	−0.0214 (12)	−0.0164 (10)	0.0025 (10)
C14	0.0565 (14)	0.0710 (16)	0.0400 (12)	−0.0198 (12)	−0.0142 (10)	−0.0021 (11)
C15	0.0767 (18)	0.084 (2)	0.0662 (16)	−0.0361 (15)	−0.0154 (14)	−0.0125 (14)
C16	0.104 (2)	0.103 (2)	0.0470 (14)	−0.0364 (19)	−0.0328 (15)	0.0091 (14)
N3	0.0607 (13)	0.0864 (16)	0.0445 (11)	−0.0141 (12)	−0.0165 (10)	0.0022 (10)
C17	0.0798 (19)	0.094 (2)	0.0447 (14)	−0.0046 (17)	−0.0231 (13)	0.0020 (14)
C18	0.085 (2)	0.0775 (19)	0.0538 (15)	−0.0176 (16)	−0.0115 (14)	0.0084 (13)
C19	0.0737 (17)	0.0765 (18)	0.0588 (16)	−0.0207 (15)	−0.0166 (13)	−0.0022 (13)
C20	0.0643 (17)	0.104 (2)	0.0547 (15)	−0.0288 (16)	−0.0183 (13)	0.0081 (14)
C21	0.0717 (18)	0.118 (3)	0.0588 (16)	−0.0306 (17)	−0.0282 (14)	0.0032 (16)

### Geometric parameters (Å, °)

**Table:**

S—C2	1.714 (2)	C10—H10A	0.93
S—C3	1.718 (2)	C11—H11A	0.93
O1—C1	1.214 (3)	C13—C14	1.508 (3)
O2—C1	1.304 (3)	C13—H13A	0.97
O2—H2A	0.82	C13—H13B	0.97
O3—C9	1.352 (3)	C14—C15	1.516 (4)
O3—C13	1.442 (3)	C14—C16	1.526 (4)
N1—C3	1.310 (3)	C14—H14A	0.98
N1—C4	1.367 (3)	C15—H15A	0.96
N2—C12	1.143 (4)	C15—H15B	0.96
C1—C2	1.485 (3)	C15—H15C	0.96
C2—C4	1.369 (4)	C16—H16A	0.96
C3—C6	1.472 (3)	C16—H16B	0.96
C4—C5	1.494 (3)	C16—H16C	0.96
C5—H5A	0.96	N3—C20	1.323 (4)
C5—H5B	0.96	N3—C19	1.330 (4)
C5—H5C	0.96	C17—C18	1.357 (4)
C6—C11	1.386 (3)	C17—C21	1.357 (5)
C6—C7	1.386 (3)	C17—H17A	0.93
C7—C8	1.391 (3)	C18—C19	1.377 (4)
C7—H7A	0.93	C18—H18A	0.93
C8—C9	1.396 (3)	C19—H19A	0.93
C8—C12	1.432 (4)	C20—C21	1.371 (4)
C9—C10	1.384 (3)	C20—H20A	0.93
C10—C11	1.382 (3)	C21—H21A	0.93
			
C2—S—C3	89.34 (11)	O3—C13—C14	107.94 (19)
C1—O2—H2A	109.5	O3—C13—H13A	110.1
C9—O3—C13	118.94 (18)	C14—C13—H13A	110.1
C3—N1—C4	111.2 (2)	O3—C13—H13B	110.1
O1—C1—O2	124.6 (2)	C14—C13—H13B	110.1
O1—C1—C2	123.2 (3)	H13A—C13—H13B	108.4
O2—C1—C2	112.2 (2)	C13—C14—C15	111.3 (2)
C4—C2—C1	129.7 (2)	C13—C14—C16	109.1 (2)
C4—C2—S	110.16 (17)	C15—C14—C16	110.7 (2)
C1—C2—S	120.2 (2)	C13—C14—H14A	108.6
N1—C3—C6	123.1 (2)	C15—C14—H14A	108.6
N1—C3—S	114.60 (16)	C16—C14—H14A	108.6
C6—C3—S	122.33 (18)	C14—C15—H15A	109.5
N1—C4—C2	114.7 (2)	C14—C15—H15B	109.5
N1—C4—C5	118.6 (2)	H15A—C15—H15B	109.5
C2—C4—C5	126.7 (2)	C14—C15—H15C	109.5
C4—C5—H5A	109.5	H15A—C15—H15C	109.5
C4—C5—H5B	109.5	H15B—C15—H15C	109.5
H5A—C5—H5B	109.5	C14—C16—H16A	109.5
C4—C5—H5C	109.5	C14—C16—H16B	109.5
H5A—C5—H5C	109.5	H16A—C16—H16B	109.5
H5B—C5—H5C	109.5	C14—C16—H16C	109.5
C11—C6—C7	117.9 (2)	H16A—C16—H16C	109.5
C11—C6—C3	121.4 (2)	H16B—C16—H16C	109.5
C7—C6—C3	120.7 (2)	C20—N3—C19	117.5 (2)
C6—C7—C8	120.5 (2)	C18—C17—C21	118.6 (3)
C6—C7—H7A	119.8	C18—C17—H17A	120.7
C8—C7—H7A	119.8	C21—C17—H17A	120.7
C7—C8—C9	120.9 (2)	C17—C18—C19	119.4 (3)
C7—C8—C12	119.5 (2)	C17—C18—H18A	120.3
C9—C8—C12	119.6 (2)	C19—C18—H18A	120.3
O3—C9—C10	125.7 (2)	N3—C19—C18	122.2 (3)
O3—C9—C8	115.8 (2)	N3—C19—H19A	118.9
C10—C9—C8	118.6 (2)	C18—C19—H19A	118.9
C11—C10—C9	119.9 (2)	N3—C20—C21	123.0 (3)
C11—C10—H10A	120.0	N3—C20—H20A	118.5
C9—C10—H10A	120.0	C21—C20—H20A	118.5
C10—C11—C6	122.2 (2)	C17—C21—C20	119.2 (3)
C10—C11—H11A	118.9	C17—C21—H21A	120.4
C6—C11—H11A	118.9	C20—C21—H21A	120.4
N2—C12—C8	178.1 (3)		
			
O1—C1—C2—C4	−0.7 (4)	C6—C7—C8—C9	−0.4 (4)
O2—C1—C2—C4	179.4 (2)	C6—C7—C8—C12	−178.2 (2)
O1—C1—C2—S	−179.5 (2)	C13—O3—C9—C10	0.5 (3)
O2—C1—C2—S	0.6 (3)	C13—O3—C9—C8	−179.5 (2)
C3—S—C2—C4	0.17 (19)	C7—C8—C9—O3	−178.6 (2)
C3—S—C2—C1	179.2 (2)	C12—C8—C9—O3	−0.8 (4)
C4—N1—C3—C6	−179.6 (2)	C7—C8—C9—C10	1.4 (4)
C4—N1—C3—S	0.1 (3)	C12—C8—C9—C10	179.1 (2)
C2—S—C3—N1	−0.1 (2)	O3—C9—C10—C11	178.9 (2)
C2—S—C3—C6	179.5 (2)	C8—C9—C10—C11	−1.0 (3)
C3—N1—C4—C2	0.1 (3)	C9—C10—C11—C6	−0.2 (4)
C3—N1—C4—C5	−179.3 (2)	C7—C6—C11—C10	1.1 (4)
C1—C2—C4—N1	−179.0 (2)	C3—C6—C11—C10	−178.8 (2)
S—C2—C4—N1	−0.2 (3)	C9—O3—C13—C14	−179.00 (19)
C1—C2—C4—C5	0.3 (4)	O3—C13—C14—C15	64.7 (3)
S—C2—C4—C5	179.2 (2)	O3—C13—C14—C16	−173.0 (2)
N1—C3—C6—C11	2.2 (4)	C21—C17—C18—C19	−0.1 (5)
S—C3—C6—C11	−177.39 (18)	C20—N3—C19—C18	0.0 (4)
N1—C3—C6—C7	−177.7 (2)	C17—C18—C19—N3	0.2 (4)
S—C3—C6—C7	2.7 (3)	C19—N3—C20—C21	−0.2 (4)
C11—C6—C7—C8	−0.8 (4)	C18—C17—C21—C20	−0.1 (5)
C3—C6—C7—C8	179.1 (2)	N3—C20—C21—C17	0.2 (5)

### Hydrogen-bond geometry (Å, °)

**Table:**

*D*—H···*A*	*D*—H	H···*A*	*D*···*A*	*D*—H···*A*
O2—H2A···N3^i^	0.82	1.79	2.611 (3)	174
C5—H5A···O1	0.96	2.52	3.055 (3)	115

Symmetry codes: (i) *x*, *y*, *z*−1.
